# C-REACTIVE PROTEIN LEVELS AMONG LGB OLDER ADULTS: INTERSECTIONAL ASSOCIATIONS OF RACE, ETHNICITY, AND EDUCATION

**DOI:** 10.1093/geroni/igae098.1201

**Published:** 2024-12-31

**Authors:** Andrew Alberth, Anyah Prasad, Amanda Collins, Alison Rataj, Heather Farmer, Jeffrey Stokes

**Affiliations:** University of Massachusetts Boston, Boston, Massachusetts, United States; University of Massachusetts Boston, Boston, Massachusetts, United States; University of Massachusetts Boston, Boston, Massachusetts, United States; University of Massachusetts Boston, Durham, New Hampshire, United States; University of Delaware, Newark, Delaware, United States; University of Massachusetts Boston, Boston, Massachusetts, United States

## Abstract

**Objectives:**

This paper assesses the intersection between sexual orientation, race, and ethnicity as they relate to CRP levels among middle-aged and older adults in the Health and Retirement Study (HRS). It will also explore how educational attainment, a commonly used demographic correlate with socioeconomic status, may differently benefit racially, ethnically, and sexually diverse middle-age and older adults.

**Methods:**

This study uses data from the 2016 Health and Retirement study. C-reactive protein levels, sexual orientation, race and ethnicity, educational attainment, and covariate variables were collected using serum samples and survey responses from 1,426 older adults enrolled in the study. Individuals were included if they had reported measures for key independent and dependent variables. Multiple imputation was used to address missing values across control variables. Associations and moderators were assessed using ordinary least squares (OLS) regression.

**Results:**

This study found lesbian, gay, and bisexual (LGB) respondents had lower CRP levels when they had higher educational attainment compared to similarly educated straight respondents. Additionally, Black LGB respondents had less inflammation than Black and straight or White and LGB respondents.

**Discussion:**

This study’s findings indicate educational attainment may differently benefit LGB older adults compared to straight older adults, and respondents who are Black and LGB experience greater protection from elevated CRP levels compared to only Black or LGB respondents. These findings suggest educational attainment may be differently used to protect against systemic inflammation among sexual minority populations. Furthermore, these results suggest additional nuance is necessary to understand how CRP levels impact diverse populations.

